# SARS-CoV-2 Exposed Mesenchymal Stromal Cell from Congenital Pulmonary Airway Malformations: Transcriptomic Analysis and the Expression of Immunomodulatory Genes

**DOI:** 10.3390/ijms222111814

**Published:** 2021-10-30

**Authors:** Andrea Valeri, Luigi Chiricosta, Agnese Gugliandolo, Mara Biasin, Maria Antonietta Avanzini, Valeria Calcaterra, Gioia Cappelletti, Stephana Carelli, Gian Vincenzo Zuccotti, Serena Silvestro, Emanuela Mazzon, Gloria Pelizzo

**Affiliations:** 1 IRCCS Centro Neurolesi “Bonino-Pulejo”, Via Provinciale Palermo, Contrada Casazza, 98124 Messina, Italy; andrea.valeri@irccsme.it (A.V.); luigi.chiricosta@irccsme.it (L.C.); agnese.gugliandolo@irccsme.it (A.G.); serena.silvestro@irccsme.it (S.S.); 2 Department of Biomedical and Clinical Sciences-L. Sacco, University of Milan, 20157 Milan, Italy; mara.biasin@unimi.it (M.B.); gioia.cappelletti@unimi.it (G.C.); gianvincenzo.zuccotti@unimi.it (G.V.Z.); gloriapelizzo@gmail.com (G.P.); 3 Cell Factory, Pediatric Hematology Oncology Unit, Fondazione IRCCS Policlinico San Matteo, 27100 Pavia, Italy; ma.avanzini@smatteo.pv.it; 4 Department of Pediatrics, Children’s Hospital “Vittore Buzzi”, 20154 Milano, Italy; valeria.calcaterra@unipv.it; 5 Pediatrics and Adolescentology Unit, Department of Internal Medicine, University of Pavia, 27100 Pavia, Italy; 6 Pediatric Clinical Research Center, Fondazione Romeo ed Enrica Invernizzi, University of Milan, 20157 Milan, Italy; stephana.carelli@unimi.it; 7 Pediatric Surgery Department, Children’s Hospital “Vittore Buzzi”, 20154 Milano, Italy

**Keywords:** mesenchymal stromal cell, congenital pulmonary airway malformations, SARS-CoV-2, transcriptomic analysis

## Abstract

The inflammatory response plays a central role in the complications of congenital pulmonary airway malformations (CPAM) and severe coronavirus disease 2019 (COVID-19). The aim of this study was to evaluate the transcriptional changes induced by SARS-CoV-2 exposure in pediatric MSCs derived from pediatric lung (MSCs-lung) and CPAM tissues (MSCs-CPAM) in order to elucidate potential pathways involved in SARS-CoV-2 infection in a condition of exacerbated inflammatory response. MSCs-lung and MSCs-CPAM do not express angiotensin-converting enzyme 2 (*ACE2*) and transmembrane serine protease 2 (*TRMPSS2*). SARS-CoV-2 appears to be unable to replicate in MSCs-CPAM and MSCs-lung. MSCs-lung and MSCs-CPAM maintained the expression of stemness markers MSCs-lung show an inflammatory response (*IL6*, *IL1B*, *CXCL8*, and *CXCL10*), and the activation of Notch3 non-canonical pathway; this route appears silent in MSCs-CPAM, and cytokine genes expression is reduced. Decreased value of p21 in MSCs-lung suggested no cell cycle block, and cells did not undergo apoptosis. MSCs-lung appears to increase genes associated with immunomodulatory function but could contribute to inflammation, while MSCs-CPAM keeps stable or reduce the immunomodulatory receptors expression, but they also reduce their cytokines expression. These data indicated that, independently from their perilesional or cystic origin, the MSCs populations already present in a patient affected with CPAM are not permissive for SARS-CoV-2 entry, and they will not spread the disease in case of infection. Moreover, these MSCs will not undergo apoptosis when they come in contact with SARS-CoV-2; on the contrary, they maintain their staminality profile.

## 1. Introduction

Coronavirus disease 2019 (COVID-19) is an infectious disease characterized by a severe acute respiratory syndrome caused by coronavirus 2 (SARS-CoV-2), a positive-sense, single-stranded RNA virus of the *Coronaviridae* family [1]. The majority of patients show mild symptoms and a suitable prognosis. However, up to 15% of patients will develop severe pneumonia, acute respiratory distress syndrome (ARDS), cardiac and renal injury, or multiorgan failure associated with the need for long-term hospitalization, ventilatory assistance, high mortality rates, and potential long-term morbidity in survivors [2].

Cell-based therapy was proposed as a treatment for respiratory system diseases, and encouraging results have been reported following mesenchymal stromal cells (MSCs) infusion [3]. Due to their capacity to produce and secrete a variety of paracrine factors and bioactive macro-molecules and their immunomodulatory effects, they are considered key players in lung tissue injury and function repair. Recently, MSC-based therapies were also introduced as a therapeutic option against severe COVID-19 [4,5,6]. Depending on the environment they encounter, MSCs release mediators with antiapoptotic, immunomodulatory, antifibrotic, chemoattractant, or proangiogenic effects, among others. In SARS-CoV-2 infection, viral particles enter target cells through an interaction of the coronavirus spike (S) protein with host cell angiotensin-converting enzyme 2 (ACE2), a proteolytic process that involves the transmembrane serine protease 2 (TMPRSS2), followed by a cascade of intracellular signaling. Interestingly, MSCs do not express ACE2 and are therefore relatively protected from infection. Additionally, it was shown that after stem cells transplantation, patients showed a significant improvement in respiratory conditions. The data also showed that peripheral lymphocyte levels increased while immune cells that secrete activated cytokines decreased. Therefore, ACE2^−^ MSCs, preventing the release of cytokines by the immune system and promoting endogenous repair due to their regenerative properties, are proposed as valid candidates for the therapy of patients with COVID-19. This encourages future investigation in order to establish the efficacy of this therapeutic modality in lung diseases [7].

MSCs are also proposed in the treatment of congenital lung disorders in pediatrics [8,9]. A therapeutic approach using stem cells was tested after repeated administration of allogenic MSCs, which induced progressive improvement of respiratory conditions in children with interstitial lung disease [9]. The regenerative process in the lung is dependent on a pool of lung MSCs that should allow lung regeneration with the maintenance of a normal architecture during the regeneration phase. However, failure in this phase has been suggested to account for the etiology of many chronic lung diseases. Moreover, in some cases, such as in congenital pulmonary airway malformations (CPAM), MSCs could be involved in tumorigenesis, and their therapeutic role is not fully elucidated. CPAM is the most frequently encountered lung lesion that results from early airway defects [10,11]. An association between CPAM and tumor has been reposted in adults and in pediatrics [11,12]. Chronic inflammation could be considered a predisposing factor toward tumorigenesis in CPAM; unveiling the interaction between the tumor, the immune system, and MSCs is crucial for understanding tumorigenesis [13]. As reported by Pelizzo [14], CPAM-derived MSCs exhibit specific features similar to tumor-derived MSCs; signals derived by chronic lung inflammation induce MSCs to alter their phenotype and immunomodulatory potential acquiring tumor-promoting or tumor-suppressing capabilities.

As reported by Stingi [15], a potential long-term oncogenic effect of SARS-CoV-2 infection could not be excluded a priori. Due to the current pandemic context, it is of great clinical importance to understand if the lung MSCs of the patients affected with CPAM, so both MSCs from perilesional or cystic origin, will be permissive for SARS-CoV-2 infection and become in such way a reservoir for virus replication influencing the malignancy risk. Moreover, the fate of these cells after SARS-CoV-2 exposure should be determined in order to understand if they will undergo apoptosis. In order to reach this aim, MSCs were harvested from pediatric patients after CPAM’s cysts surgery resection, from perilesional tissue (MSCs-lung) and cystic tissue (MSCs-CPAM), and exposed in vitro to SARS-CoV-2. Their transcriptomic profile was further analyzed.

## 2. Results

In vitro expanded cells from the patient’s pulmonary tissue showed plastic adherence, spindle shape morphology, and cell surface markers positivity for CD73, CD90, CD105, and HLA-I and negativity for CD34, CD45, CD14, CD31, and HLA-DR. After in vitro culture in the presence of appropriate stimuli, cells showed osteogenic differentiation potential but lacked adipogenic differentiation capacity. Cells entered senescence phases at passage 14 (P14). Considering these characteristics, we defined these cells as MSCs-lung [16].

Both the MSCs-CPAM and the MSCs-lung were exposed to SARS-CoV-2 in an in vitro infection assay. Notably, analyses performed on the cell culture supernatant over time (24–72 hpi) did not show an increase in viral replication, assessed by means of real-time PCR, neither in MSCs-CPAM nor in MSCs-lung [17]).

Transcriptomic analyses were performed by comparing SARS-CoV-2 exposed and unexposed samples (mock) in both MSCs-CPAM and MSCs-lung.

**Figure 1:**
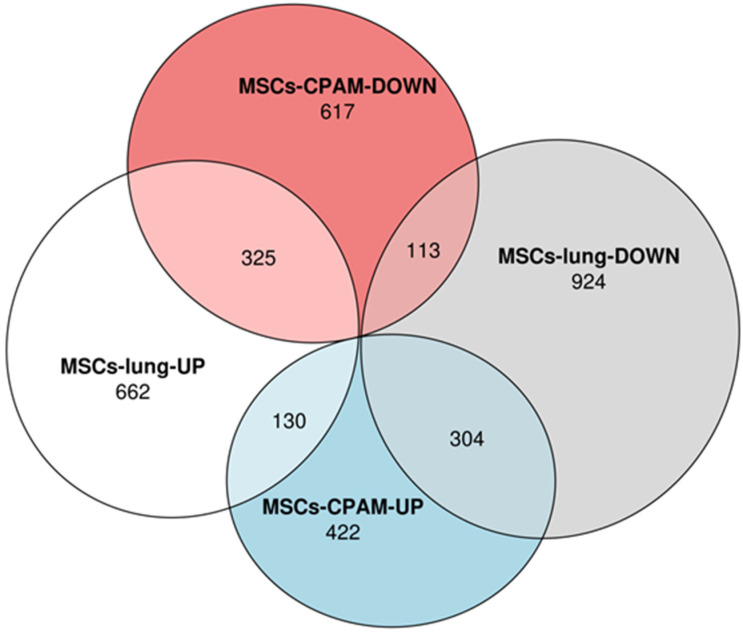
Distribution of differentially expressed genes (DEGs) in MSCs-lung or in MSCs-CPAM conditions. The Euler diagram shows how up and downregulated DEGs were distributed in MSCs-lung or in MSCs-CPAM after SARS-CoV-2 exposure. Particularly, the deregulation followed the same trend for 438 DEGs (130 were upregulated and 113 were downregulated). Conversely, 304 DEGs were downregulated in MSCs-lung and upregulated in MSCs-CPAM, and the opposite trend is observed for 325 DEGs.

Transcriptomic analyses performed on MSCs-lung samples reveal that 1117 differentially expressed genes (DEGs) were upregulated while 1341 were downregulated after SARS-CoV-2 exposure. On the other hand, the MSCs-CPAM showed 1911 deregulated DEGs, among which 856 were up and 1055 downregulated after SARS-CoV-2 exposure. The Euler diagrams in Figure 1 depict how the up and downregulated DEGs were distributed in MSCs-lung or in MSCs-CPAM conditions. Interestingly, 130 DEGs were upregulated both in MSCs-lung and in MSCs-CPAM, while 113 were downregulated in both groups after SARS-CoV-2 exposure. On the other hand, 304 DEGs downregulated in MSCs-lung were upregulated in MSCs-CPAM, and the opposite situation occurred for 325 DEGs.

The steamness profile of both types of MSCs was evaluate also after SARS-CoV-2 exposure, as shown in Table 1. In order to confirm MSCs-lung as proper control, we also included MSCs from tracheal aspirates of intubated preterm newborns without bronchopulmonary dysplasia (MSCs-TA), confirming their stemness.

**Figure 2:**
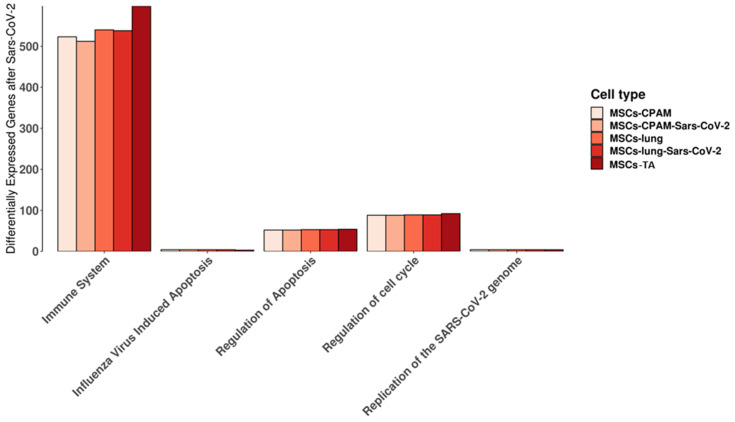
Reactome pathways in which the genes expressed in MSCs-lung or in MSCs-CPAM before and after SARS-CoV-2 exposure, MSCs-TA are expressed. For each experimental group, the plot shows the amount of genes included in the pathway. “Immune system” stands for “cytokine signaling in immune system” while “regulation of cell cycle” is “regulation of mitotic cell cycle” pathways. No differences were highlighted in “influenza virus-induced apoptosis” and in “replication of the SARS-CoV-2 genome”. Limited differences were instead observed in the other pathways.

We used the Reactome database to highlight the pathways in which the genes expressed in MSCs-lung, MSCs-lung-SARS-CoV-2 exposed, MSCs-CPAM or MSCs-CPAM-SARS-CoV-2 exposed, MSCs-TA were included. In detail, we observed the behavior of the experimental groups in the “cytokine signaling in immune system”, “regulation of mitotic cell cycle”, “replication of the SARS-CoV-2 genome”, “regulation of apoptosis”, and “influenza virus-induced apoptosis” pathways that are shown in Figure 2. Interestingly, we did not observe any difference in “influenza virus-induced apoptosis” and in “replication of the SARS-CoV-2 genome” and only limited dissimilarities in the other pathways.

Given the role of Notch as a bridge between viral infections and immune response, we inspected the Notch signaling pathway. As represented in Table 2, we found in MSCs-lung the activation of *NOTCH3*, of the ligand *DDL4* and the downregulation of the inhibitors *DVL2* and *NUMBL*. Conversely, in MSCs-CPAM, *NOTCH3* was not deregulated, and the cleaver enzyme *ADAM17* was downregulated while *DVL1* and *NUMBL* inhibitors were upregulated. Notch seems to promote its activity through the non-canonical pathway. Indeed, *RBPJ* should be activated after the internalization of the Notch intracellular domain. Nevertheless, it was downregulated in MSCs-lung and upregulated in MSCs-CPAM. The hyphen symbol in the table indicated no DEG in the experimental group. All the values are rounded to the second decimal digit.

To establish the different roles of Notch signaling in MSCs-lung and MSCs-CPAM after SARS-CoV-2 exposure, we inspected the related pathways on KEGG “MAPK signaling pathway”, “NF-kappa B signaling pathway”, “toll-like receptor signaling pathway”, “cell cycle”, and “apoptosis”. Table 3 included the DEGs that were linked to the Notch response.

**Figure 3:**
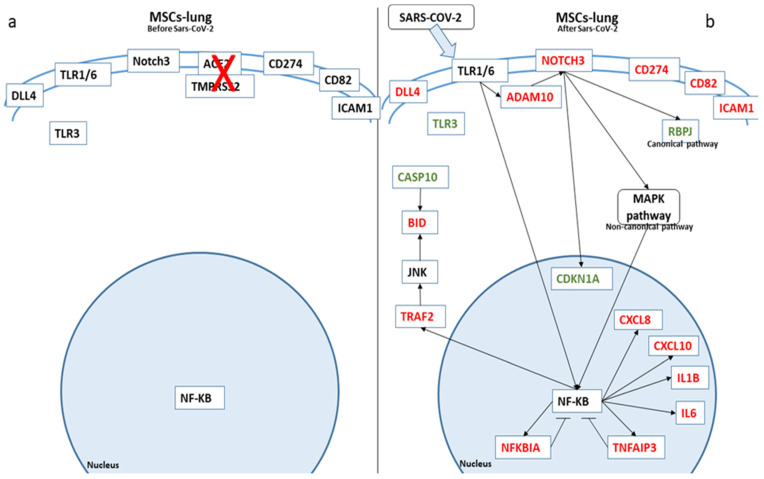
Biological representation of genes involved in Notch signaling before (**a**) and after (**b**) SARS-CoV-2 exposure in MSCs-lung. We observed the differences in MSCs-lung before and after SARS-CoV-2 exposure. The arrows show the biological activation of the genes. The genes represented in red are upregulated in MSCs-lung after SARS-CoV-2 exposure. Conversely, the genes in green are downregulated. Whereby the genes are depicted in black, no statistical difference was observed.

**Figure 4:**
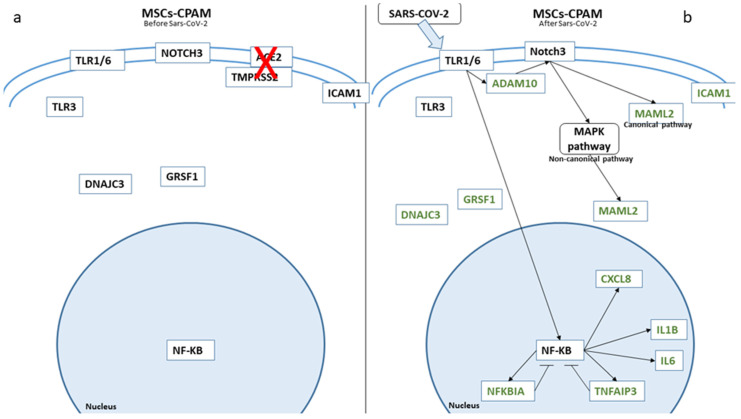
Biological representation of genes involved in Notch signaling before (**a**) and after (**b**) SARS-CoV-2 in MSCs-CPAM. We observed the differences in MSCs-CPAM before and after SARS-CoV-2 contact. The arrows show the biological activation of the genes. The genes in green are downregulated. Whereby, for genes depicted in black, no statistical difference was observed.

In Figure 3, we depicted the biological role of these genes in MSCs-lung before and after SARS-CoV-2 exposure, while in Figure 4, we report the role in MSCs-CPAM.

**Figure 5:**
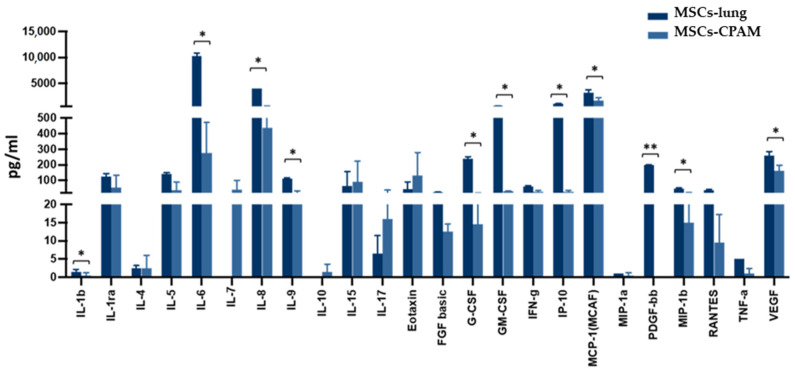
Cytokines and chemokines release in the secretome of SARS-CoV-2 exposed MSCs-lung and MSCs-CPAM 72 hpi. The levels of several proinflammatory cytokines and chemokines, assessed by multiplex ELISA, were significantly higher in MSCs-lung compared to MSCs-CPAM following viral exposure. The mean values of the two patients ± SE are reported for MSCs-lung and MSCs-CPAM. * = *p* < 0.05, ** = *p* < 0.01.

The activation of the inflammatory response in SARS-CoV-2 exposed MSCs-lung was further confirmed by analyzing the cell culture supernatant 72 hpi. Indeed, as shown in Figure 5, following SARS-CoV-2 exposure, MSCs-lung released higher quantities of several proinflammatory cytokines and chemokines in their secretome compared to MSCs-CPAM, reaching statistical significant differences for: IL-1 (*p* < 0.04), IL-6 (*p* < 0.02), IL-8 (*p* < 0.02), IL-9 (*p* < 0.04), G-CSF (*p* < 0.02), GM-CSF (*p* < 0.02), CXCL10 (*p* < 0.04), CCL2 (*p* < 0.02), CCL4 (*p* < 0.05), CCL5 (*p* < 0.04), PDGF (*p* < 0.01), and VEGF (*p* < 0.03).

## 3. Discussion

CPAM is a congenital disease characterized by abnormal airway defects during fetal lung development, and it is characterized by cyst formation. Today, surgery remains the solution in symptomatic lesions and usually is performed in the first year of life [18]. The management of asymptomatic infants remains controversial [19,20,21]; the long-term risk of infection and potential malignancy development are the main arguments in favor of resection during infancy [22,23]. MSCs seem to be crucial players in the microenvironment and are closely linked to the regulation of tumor survival, growth, and progression [14].

Since their identification, MSCs represent a promise for a large variety of diseases since they are usually harvested from the same patient to avoid rejection from the host. They may differentiate into various tissues and secrete therapeutic factors, from growth factors to assist regeneration to immunomodulatory factors that alter the immune response [24].

In the current pandemic situation, it is important to understand whether MSCs could be involved in the SARS-CoV-2 infection, especially the response in patients affected with pathological pulmonary congenital and acquired condition and primary respiratory failure. In order to evaluate if MSCs-lung and MSCs-CPAM can sustain viral replication and if they undergo apoptosis after SARS-CoV-2 exposure, next-generation sequencing (NGS) analysis was performed.

To ensure the reliability of MSCs-lung as a control for its experimental set, a confrontation between the main genes of the pathway inspected was performed using MSCs-TA as external control, and the difference in genes will be discussed in their section during the discussion. Obvious differences due to the different origins of the cells did not change the meaning of the results, so MSCs-lung is considered proper control for its experimental group.

To answer the question of whether MSCs-lung and MSCs-CPAM can be infected with SARS-CoV-2 and spread the infection, the expression of the known genes involved in SARS-CoV-2 infection was assessed. It is widely known that SARS-CoV-2 mainly uses ACE-2 receptors to enter the cell, and much research focused on its role in the pathogenesis of the COVID-19 infection [25]. Along with ACE-2, also TMPRSS2 is used by SARS-CoV-2 as spike protein priming, and its block is proved to be efficient in stopping the infection [26]. It is known that MSCs are negative for both *ACE2* and *TMPRSS2* genes, suggesting that there is no likelihood of viral infection in the cells [7]. Additionally, an experiment with the co-culture of MSCs and infected Caco-2 cells revealed that MSCs were resistant to infection under stable and inflammatory conditions [27]. According to the previous evidence, neither *ACE2* nor *TMPRSS2* expression was found in the examined cells, independently from their origins. Indeed, both types of cells are immune to SARS-CoV-2 entry from the ACE2-dependent pathway.

However, SARS-CoV-2 was found to be capable of using receptors independent of ACE2 to penetrate the plasma membrane. In our recent work, the role of Toll-like receptors (TLRs) in SARS-CoV-2 infection was elucidated in HCN-2 cells. In this study, HCN-2 cells were infected by SARS-CoV-2 in vitro, and the effect of the infection was assessed 6 days later. We found increased expression of *TLR1*, *TLR4*, and *TLR6* [28]. This correlates with the hypothesis of the role of TLR1/2 and TLR2/6 in the trafficking of S spike protein from the cell membrane to the intracellular environment [29]. Another hint about the capacity of TLRs to mediate the entry of viruses in the cells that assume the capacity of TLRs to recognize the virus comes from another study, where the Dengue virus showed increased NF-kB expression via TLR2/6 recognition [30].

The expression of *TLR 1* and *6* was very low in both MSC-lung and MSCs-CPAM and, after exposure to SARS-CoV-2, we did not observe a significant variation in *TLR1* and *TLR6* expression values, while in a condition of active infection and virus replication, these receptors were found to be significantly upregulated.

Two different scenarios followed viral exposure in our experimental setting.

MSCs-lung augmented their level of proinflammatory cytokines; *IL6*, *IL1B*, *CXCL8*, and *CXCL10* expression was, indeed, significantly increased, displaying an expression profile mirroring the one observed in HCN2-SARS-CoV-2 infected cells. Such finding was further confirmed by analyzing the secretome of SARS-CoV-2 exposed MSCs-lung, which was significantly enriched in proinflammatory cytokines and chemokines. The level of IL6, IL1B, IL8, and IP10 resulted significantly increased in MSCs-lung compared to MSCs-CPAM after exposure to SARS-CoV-2. The concept of inflammation due to SARS-CoV-2 is not new. Several studies have, since the beginning of the pandemic, unveiled the increase in proinflammatory cytokines in SARS-CoV-2 infection [31]. They have different functions. Regarding inflammation, IL6 acts on plasma cells and induces antibody production, promoting CD4-positive T cells along with IL21; moreover, it inhibits the differentiation of Th1 cells while it stimulates the differentiation of Th2, as reviewed [32]. IL1B, during acute inflammation, stimulates the differentiation of monocytes in dendritic cells into M1 macrophages and increases the proliferation of B-cells [33]. IL8 is chemotactic for almost all the migratory immune cells, and, in mice that overexpressed IL8, it was found an increased level of circulating neutrophils [34]. IP10 has a role in the internalization of CXCR3 and thus promotes the activity of effector T cells, while it can induce apoptosis as well as regulate cell proliferation [35].

The infection also modifies the expression of MAPK cascade in HCN-2, but in our MSCs, *MAPK1* and *MAPK3* levels were decreased, and JNK, p38, and *ISG15* did not change their values.

*DDX58*, a sensor of viral RNA, is not increased either. *DDX58*, also known as RIG-1, has been proven to play an important role as a viral suppressor in A549 cells, where its knockdown significantly increases viral replication [36]. Its constant level, before and after the infection, suggests that there was no need for an upregulation because SARS-CoV-2 failed to enter or was unable to replicate in MSCs-lung.

To exclude an active replication from the virus, *DNAJC3* and *GRSF1* were evaluated: *DNAJC3* encodes for the protein p58INK, and it has been shown to be active during influenza virus replication. Moreover, cells lacking p58INK showed a reduced level of viral replication, as shown in an experiment with MEF cells derived from P58-null mice, where the viral protein synthesis decreased of two-folds compared to the MEF from WT mice [37]. *GRSF1* seems to play a role during viral RNA translation. *GRSF1* depletion in HeLa cells was associated with a 60% reduction in translational capacity, a function restored after *GRSF1* reconstitution [38]. The same trend is replicated in the confrontation between MSCs-TA and MSCs-lung after SARS-CoV-2 exposure, confirming no viral replication. None of these genes were upregulated in MSCs-lung.

*CREBBP*, known as CBP, was found downregulated in MSCs-lung while remaining constant in MSCs-CPAM. CBP has several functions in the cell since it is a transcriptional co-activator that regulates the expression of different genes: even if the specific molecular mechanism was not fully elucidated, it was reviewed its role in cell cycle progression as a substrate of phosphorylation by CDK/CyclinE [39], but it was also suggested to have a role in the viral replication. Briefly, association with CBP and Tat protein of HIV-1 virus was proofed by co-immunoprecipitation of both proteins together in HL3T1 cells; moreover, in the same experiment, it was shown that Tat protein increases the expression of CBP, recruiting it to the LTR of the viral genome [40]. Its downregulation in MSCs-lung suggests once more that probably no viral replication is active. It is found upregulated in the comparison between MSCs-TA and MSCs-lung after SARS-CoV-2 exposure, but since MSCs-TA are taken from neonatal patients, and CPB plays a different role in lung development, its expression is too prone for variation to be taken into consideration in this comparison [41].

*DTX3L*, in association with *PARP9*, can have antiviral effects by mediating the ubiquitination of viral proteases. In an experiment with U3A cell type, bearing different combinations of *PARP9* and *DTX3L* mutation, the presence of *DTX3L* was enough to decrease the amount of EMCV 3C; moreover, *DTX3L* co-localized with the viral protease in EMCV-infected U3A cells [42]. *DTX3L* was significantly reduced in MSCs-lung, while it maintains its value in MSCs-CPAM and MSC-TA confrontation, suggesting that there is no need for protease degradation and sustaining the previous hint regarding no active viral replication.

Focusing on the other cell type, following SARS-CoV-2 exposure MSCs-CPAM, as mentioned before, reduced *IL6*, *IL1B*, *CXCL8*, and *CXCL10* expression, suggesting a control over the inflammation process. This result was further confirmed by unaltered MAPKs genes (*MAPK1*, *MAPK3*, JNK, and *MAPK14*). These results further reinforced the assumption that SARS-CoV-2 was not able to enter MSCs-CPAM using an ACE2-independent way. To exclude a silent entry of the virus and its capacity to not trigger any reaction while replicating, *DNAJC3* and *GRASF1* levels were evaluated, and they resulted in a decrease.

Taken together, these findings suggest that viral replication did not occur in MSCs-lung and MSCs-CPAM. These results were predicted by the analysis reported in Figure 2, where the number of genes involved in the replication of the SARS-CoV-2 genome pathway did not differ before and after SARS-CoV-2 exposure in both cell types.

The inflammatory reaction in MSCs-lung is likely due to the interaction of SARS-CoV-2 with TLRs, whose cascade culminates with the activation of NF-κB: upon activation, NF-κB translocates into the nucleus and binds to DNA to promote the transcription of different genes, proinflammatory cytokines among them. *NUMBL*, encoding for Numb-like protein, is known to be a negative regulator of NF-κB: in an experiment of osteoclastic differentiation, using cells isolated from the bone marrow of RElA-luc reporter mice, it was clear that NUMBL protein decreased RelA levels, a subunit of NF-κB, after RANKL treatment [43]. So, it is coherent to find *NUMBL* downregulated in MSCs-lung, where we suppose the activation of NF-κB, and found it upregulated in MSCs-CPAM, where there should not be activation of NF-κB. However, increased levels of *NFKBIA* and *TNFAIP3*, two repressors of NF-κB, were found. This may indicate a balance between activation and suppression that results in no variation of gene expression. In the comparison between MSCs-TA and MSCs-TA, *NUMBL* was found upregulated, but it was also found an upregulation of *NFKB1*, a subunit of NF-κB: this confirms the activation of NF-κB signaling following SARS-CoV-2 exposure and the activation also of a compensatory mechanism to balance this activation. Indeed, also in the comparison between MSCs-TA and MSCs-lung after SARS-CoV-2 exposure is reported the upregulation of proinflammatory signal.

However, TLRs can also interact with *ADAM10*, the metalloprotease protein responsible for the cleavage of Notch intracellular domain, as was shown in an experiment of activated macrophages, where the signaling of TLRs correspond to an increase in *ADAM10* [44]. *ADAM10* was indeed upregulated in MSCs-lung cells after SARS-CoV-2 exposure. Moreover, *ADAM17* and *ADAM10* are downregulated after SARS-CoV-2 infection in MSCs-CPAM, and both play a role in the Notch signaling [45].

In our experiment, in MSCs-lung, the level of *NOTCH3* was upregulated, while in MSCs-CPAM, there were no effects on *NOTCHs* genes. Upon activation, Notch signaling can follow different pathways. Given that there is no variation of the genes of the canonical Notch pathway *HES1* and *HEYL* and that *RPBJ* value is reduced, it is correct to think of a non-canonical pathway. It is important to mention that activation of NOTCH signaling also correlates with IL-6 [46] and GM-CSF regulation [47]. Notch is also associated with NF-κB activation, and its role in inflammatory response was documented while blocking the Notch signaling correspond to reduced inflammation and impaired NF-κB activation [48]. Another work in activated macrophages connects TLRs action, Notch3 activation, and NF-κB activity, and the selective silencing of Notch3 inhibits the activity of NF-κB [49]. Translating this evidence in our study, ELISA tests on the conditioned medium highlight the difference between the secretion of IL-6 and proinflammatory cytokines between MSCs-lung and MSCs-CPAM, while the first group showed increased *NOTCH3* value and the second suggest a block of Notch signaling. Indeed *NCOR2*, a transcription repressor, was found downregulated in MSCs-lung and upregulated in MSCs-CPAM. In a study about the development of zebrafish, *NCOR2* was found to inhibit Notch signaling by giving Notch inhibitor DAPT to morphant *NCOR2* embryos and rescuing, in such a way, runx1 expression [50]. The evidence of its downregulation in MSCs-lung supports the activation of Notch signaling, while its overexpression in MSCs-CPAM reinforces the inactivity of the Notch pathway. Even if in MSCs-TA and MSCs-lung, after SARS-CoV-2 exposure, the level of *NCOR2* was increased, it is worth mentioning that also *RBPJ* was increased: this indicates that the canonical Notch signaling pathway may be activated, with an increase in *NCOR2* as compensatory.

*NUMB* expression is downregulated in MSCs-CPAM and, even if it has been reported as a Notch repressor, other evidence showed *NUMB* to play a role in the stabilization of the intracellular part of the Notch1 receptor (N1ICD). The knockdown of NUMB in HeLa cells leads to a reduction in endogenous cleaved Notch1 and N1ICD [51]. Our interpretation of its reduction in MSCs-CPAM is that, since there is no overexpression of *NOTCHs*, the repressing activity is not needed, and so *NUMB* is downregulated.

Since Notch signaling has a role in the cell cycle, as shown in an experiment in activated T cells [52], we investigate if the activation of *NOTCH3* results in a variation of *CDK4* or *CDK6*. There was no variation of *CDK4* and *CDK6* genes, and their repressor p21, encoded by the gene *CDKN1A*, shows a reduced value, suggesting that there is no repression in the normal flow of the cell cycle. No variation was also found in *CDK4* and *CDK6* genes in MSCs-CPAM.

*TRAF2* is another gene that can be transcripted by activation of NF-κB, and it is known to interact with JNK for anti-apoptotic purposes, as shown in an experiment where *TRAF2* was selectively inhibited in lymphocytes, and they showed an increased death ratio, compared to the controls [53]. Even if the level of JNK is not different before and after the infection, there is a decrease in *CASP10* value, encoding for Caspase-10. Caspase-10 plays a role in the apoptosis cleaving Bid, as is clear from an experiment using Caspase 8-deficient Jurkat cells, where Bid was cleaved in a Caspase-10 dose-dependent manner [54].

Taken together, these results suggest that SARS-CoV-2 exposure did not seem to affect the cell cycle or apoptosis in MSCs-lung. No variation in the examined cell cycle genes was also found in MSCs-CPAM. This correlates with Figure 2, where no difference was clear before and after SARS-CoV-2 exposure in regulation of apoptosis and cell cycle in both types of cells, in particular the one dedicated to influenza virus-induced apoptosis.

Mesenchymal cells are also used in regenerative medicine for their immunomodulatory function [3], so the expression of the immunomodulatory protein at the cell surface was inspected.

MSCs-lung showed upregulation of *CD274*, encoded for PD-L1. The axis PD1/PDL1 plays a role in the increase in the expression of *FOXP3* in Treg cells and promotes the conversion of Th in Treg [55]. *ICAM1*, upregulated in MSCs-lung, plays a role in the recruitment of neutrophils, as shown in ICAM1-null mice, where the recruitment of neutrophils was reduced by 54% [56]. Returning to the Notch pathway, *DLL4* has been overexpressed in MSCs-lung, and it is known to promote differentiation of Th17 cells, as discussed [57].

Neither *CD274* nor *DLL4* was upregulated in MSCs-CPAM, while *ICAM1* expression was downregulated.

Secretome analysis unveils the presence of other immunomodulatory molecules secreted in higher quantities by MSCs-lung compared to MSCs-CPAM after SARS-CoV-2 exposure. IL9 has different functions, as reviewed [58]. However, the proliferative effects on T cells are important in our context; G-CSF is a growth factor, and its role in the differentiation, as well as in the survival of neutrophilic granulocytes, was reviewed. Indeed, mice lacking G-CSF or its receptor show a significantly reduced amount of mature and circulating neutrophils [59]. Regarding GM-CSF, a review explains its properties, among them the capacity to regulate T cells and, in particular, overexpression of GM-CSF lead to macrophages accumulation and damage to other tissues, so it is clear its role as a proinflammatory cytokine [60]. MCP-1, encoded by the gene *CCL2*, and MIP-1B, encoded by the gene *CCL4*, are both chemokines involved in the recruitment and differentiation of macrophages: a study conducted on colon-rectal cancer patients correlates CCL4 with the differentiation of monocytes in M2 macrophages, while CCL2 is already correlated with the infiltration degree of macrophages CD68-positive [61].

Since MSCs-lung and MSCs-CPAM did not undergo apoptosis, they may follow differentiation pathways, so the markers of staminality were assessed. As reported in Table 1, both cell types, before and after SARS-CoV-2 exposure, maintained the expression of the MSCs markers.

Taken together, these results suggest that SARS-CoV-2 exposure was unable to alter the stemness profile of MSCs-lung and MSCs-CPAM. On the contrary, MSCs-lung seems to respond to the viral presence with an increase in immunomodulatory function, while MSCs-CPAM does not sensible vary their immunomodulatory profile.

The small number of subjects (n = 2) represent a clear limitation of our study. CPAM, despite being the most common form of congenital cystic lung disease, has an incidence ranging from 1 in 10,000 to 1 in 35,000 births. This makes it difficult to recruit a larger patient cohort.

## 4. Materials and Methods

### 4.1. Patients

Two male infants diagnosed with CPAM were admitted to the surgical department for elective surgery. Microscopically, the lesion was diagnosed as type II CPAM by Stocker’s classification. After written informed consent was obtained from the parents, a small portion of the pulmonary tissue destined for histological analysis was used for MSC expansion. One sample was derived from the cyst wall (MSCs-CPAM), while the other one was taken from a “healthy” section (called MSCs-lung). The study was performed according to the Declaration of Helsinki and with the approval of the Institutional Review Board of the Children’s Hospital “G. Di Cristina” (registry number 87 Civico 2017). Informed written consent was obtained from the parents and/or legal guardian after receiving information about the study.

### 4.2. Isolation, Culture, and Characterization of MSCs

The MSCs were obtained from pulmonary tissue of two male infants diagnosed with CPAM and submitted to elective surgery. Microscopically, the lesion was diagnosed as type II CPAM by Stocker’s classification. After written informed consent was obtained from the parents, a small portion of the pulmonary tissue destined for histological analysis was used for MSC expansion. One sample was derived from the cyst wall (MSCs-CPAM), while the other one was taken from a “healthy” section (called MSCs-lung). Isolation and expansion of MSCs from pulmonary tissue were performed as previously described [62]. Briefly, the pulmonary samples were treated with collagenase type II to obtain cell suspension that was then plated in a culture flask at a density of 160,000/cm^2^. Cultures were maintained at 37 °C, 5% CO_2_, replacing medium twice a week. When ≥80% confluence was reached, cells were harvested with Trypsin EDTA and replated for expansion at a density of 4000 cells/cm^2^. MSCs-CPAM and MSCs-lung were characterized following the criteria defined by Dominici et al. [16]. Cell surface markers were evaluated by flow cytometry, and differentiation capacity toward adipocytes and osteocytes was assessed as previously described [14].

### 4.3. Virus

SARS-CoV-2 (Human 2019-nCoV strain 2019-nCoV/Italy-INMI1, Rome, Italy) was provided by the European Virus Archive goes Global (EVAg) through the National Institute for Infectious Diseases “Lazzaro Spallanzani” IRCCS organization. The virus was expanded on Calu-3 cells (ATCC^®^ HTB-55™, Manassas, VA, USA), and viral titers were determined by TCID_50_ endpoint dilution assay. Briefly: serial 10-fold dilutions, from 10^6^ to 10^−4^ TCDI_50_/mL (50 μL), were plated onto 96-well plates, incubated at 37 °C in 5% CO_2_, and checked daily to monitor the virus-induced cytopathic effect. Seventy-two hours post infection (hpi) viral titer was determined as previously described [63]. All the experiments with the SARS-CoV-2 virus were performed in a BSL3 facility.

### 4.4. In Vitro MSCs SARS-CoV-2 Infection Assay

MSCs were cultured in DMEM (Euroclone, Milan, Italy) with 10% FBS medium and 100 U/mL penicillin and 100 μg/mL streptomycin in a 24-well plate. DMEM containing 100 U/mL penicillin and 100 µg/mL streptomycin was used as inoculum in the mock-infected cells. Cell cultures were infected with 1 multiplicity of infection (MOI) and incubated at 37 °C and 5% CO_2_. After 3 h, cells were washed two times with lukewarm PBS and refilled with the proper growth medium (10% FBS). Optical microscope observation (ZOE™ Fluorescent Cell Imager, Bio-Rad, Hercules, CA, USA) was performed daily to investigate the cytopathic effect. SARS-CoV-2 RNA was extracted from 24, 48, and 72 hpi supernatant using the Maxwell^®^ RSC Instrument with Maxwell^®^ RSC Viral Total Nucleic Acid Purification Kit (Promega, Fitchburg, WI, USA). Viral RNA was reverse transcribed in a single-step RT-qPCR (GoTaq 1-Step RT-qPCR; Promega, Fitchburg, WI, USA) on a CFX96 instrument (Bio-Rad, Hercules, CA, USA) using primers specifically designed to target two regions of the nucleocapsid (N1 and N2) gene of SARS-CoV-2 (2019-nCoV CDC qPCR Probe Assay emergency kit; IDT, Coralville, IA, USA), together with primers for the human RNase P gene. Viral copy quantification was assessed by creating a standard curve from the quantified 2019-nCoV_N-positive Plasmid Control (IDT, Coralville, IA, USA).

### 4.5. MSC-Lung External Control Group

Along with our control group MSCs-lung and MSCs-CPAM, we collected a data set of mesenchymal stromal cells isolated from 10 preterm newborns through tracheal aspirate-derived technique. The data set identified as PRJNA418413 [64] is stored on the Gene Expression Omnibus [65] repository of the National Center for Biotechnology Information (NCBI, Bethesda, MD, USA) [66]. From the whole repository, we analyzed only the newborns that were identified as “No Bronchopulmonary Dysplasia”.

### 4.6. RNA-Seq Analysis

For the preparation of the library, we used the TruSeq RNA Exome protocol (Illumina, San Diego, CA, USA) and the Illumina MiSeq instrument for library sequentiation. We took advantage of the fastQC software (version 0.11.5, Babraham Institute, Cambridge, UK.) to confirm the parameters of the raw data in Fastq format. With Trimmomatic (version 0.38, Usadel Lab, Aachen, Germany) [67], we removed the adapters and the base with low-quality scores. Then, we aligned the reads with spliced transcripts alignment to a reference (STAR)RNA-seq aligner (version 2.7.3a, New York, NY, USA) [68] choosing the human reference genome version GRCh38, and we performed the transcripts count with the python package htseq-count (version 0.6.1p1, European Molecular Biology Laboratory (EMBL), Heidelberg, Germany) [69]. The analysis of the differentially expressed genes (DEGs) was computed through the package DESeq2 of Bioconductor [70] in R (version 3.6.3, R Core Team). We used the post-hoc Benjamini–Hochberg method, and we kept as significant only the DEGs whose q-value was not higher than 0.05. We did not use any fold change cut-off.

### 4.7. Cytokine and Chemokine Measurement by Multiplex Assay

The concentration of cytokines/chemokines was assessed in cell culture supernatants 72 hpi by using immunoassays formatted on magnetic beads (Bio-Plex Pro Human Cytokine 27-plex Assay #M500KCAF0Y) (Bio-Rad, Hercules, CA, USA), according to manufacturer’s protocol via Luminex 100 technology (Luminex, Austin, TX, USA). A total of 0 pg/mL is attributed to values below the limit of detection.

## 5. Conclusions

Given the methodological limitations, the results of these studies revealed that MSCs-lung and MSCs-CPAM are not permissive for SARS-CoV-2 infection, so they do not represent a reservoir for viral replication inside CPAM patients lungs. The absence of apoptosis markers in both cells populations ensures their survival in the case of COVID-19. Moreover, they both maintain their staminality.

The variation of MSCs-lung in their immunomodulatory profile after SARS-CoV-2 exposure may serve as a base for further in vivo experiments to understand their potential in regenerative medicine.

## Figures and Tables

**Table 1:** Expression values of MSCs markers in MSCs-CPAM or MSCs-lung before and after SARS-CoV-2 exposure and MSCs-TA.

Genes	MSCs-Lung	MSCs-Lung Infected	MSCs-CPAM	MSCs-CPAM Infected	MSCs-TA
*NT5E*	4041.59	4097.82	1838.26	1560.99	4942.13
*THY1*	2641.86	2736.23	1092.17	1094.85	2940.66
*ENG*	1880.03	1728.19	1219.84	1019.38	575.79
*HLA-A*	7579.78	7565.95	3017.68	3353.59	1914.89
*HLA-B*	5677	6142.11	2011.94	2213.41	1209.48
*HLA-C*	4216.12	4170.44	1555.12	1642.62	656.22

**Table 2:** Fold change of differentially expressed genes (DEGs) involved in KEGG Notch signaling pathway in MSC-TA and MSCs-lung after SARS-CoV-2 exposure, MSCs-CPAM or MSCs-lung after SARS-CoV-2 exposure.

Gene	Log_2_ Fold Change MSCs-TA-Lung Exposed	Log_2_ Fold Change MSCs-Lung	Log_2_ Fold Change MSCs-CPAM
*ADAM17*	1.05	-	−1.56
*CREBBP*	3.24	−0.36	-
*DLL4*	-	5.09	-
*DTX3L*	-	−1.44	-
*DVL1*	-	-	1.56
*DVL2*	0.8	−0.65	-
*HDAC2*	0.87	-	−0.72
*JAG1*	3.33	0.4	1.59
*MAML2*	1.81	-	−1.06
*MAML3*	2.76	0.73	-
*NCOR2*	1.47	−0.59	0.71
*NOTCH3*	1.97	1.33	-
*NUMB*	-	-	−0.97
*NUMBL*	0.85	−0.75	1.61
*RBPJ*	0.55	−1.03	1.17
*TLE3*	-	-	1.03

**Table 3:** Fold change of differentially expressed genes (DEGs) triggered by Notch activation in MSC-TA and MSCs-lung after SARS-CoV-2 exposure, MSCs-CPAM or MSCs-lung after SARS-CoV-2 exposure.

Gene	Log_2_ Fold Change MSCs-TA-Lung Exposed	Log_2_ Fold Change MSCs-Lung	Log_2_ Fold Change MSCs-CPAM
*DLL4*	-	5.09	-
*CD274*	−2.38	1.77	-
*CD82*	2.48	1.56	-
*ICAM1*	1.65	1.18	−1.55
*TNFAIP3*	3.29	2.18	−2.67
*NFKB1*	1.13	-	-
*NFKBIA*	3.41	0.90	−1.86
*TRAF2*	-	1.38	-
*ADAM10*	1.53	0.35	−0.49
*IL6*	2.48	2.22	−2.53
*IL1B*	5.47	3.25	−10.39
*CXCL8*	6.07	6.25	−7.63
*CXCL10*	-	7.42	-
*CASP10*	-	−2.23	-
*CDKN1A*	-	−0.58	-
*BID*	-	1.00	−
*DNAJC3*	−1.35	-	−0.95
*GRSF1*	−0.3	-	−1.02
*MAPK10*	-	-	−2.98

## Data Availability

The data presented in this study are openly available in the NCBI Sequence Read Archive at BioProject accession number PRJNA752960.
